# Improved Agricultural Field Segmentation in Satellite Imagery Using TL-ResUNet Architecture

**DOI:** 10.3390/s22249784

**Published:** 2022-12-13

**Authors:** Furkat Safarov, Kuchkorov Temurbek, Djumanov Jamoljon, Ochilov Temur, Jean Chamberlain Chedjou, Akmalbek Bobomirzaevich Abdusalomov, Young-Im Cho

**Affiliations:** 1 Department of Computer Engineering, Gachon University, Sujeong-Gu, Seongnam-Si 461-701, Gyeonggi-Do, Republic of Korea; 2 Department of Computer Systems, Tashkent University of Information Technologies named after Muhammad Al-Khwarizmi, Tashkent 100200, Uzbekistan; 3 Institute of Smart Systems Technologies, University of Klagenfurt, 9020 Klagenfurt, Austria

**Keywords:** image segmentation, agriculture, satellite imagery, deep learning, UNet architecture, transfer learning

## Abstract

Currently, there is a growing population around the world, and this is particularly true in developing countries, where food security is becoming a major problem. Therefore, agricultural land monitoring, land use classification and analysis, and achieving high yields through efficient land use are important research topics in precision agriculture. Deep learning-based algorithms for the classification of satellite images provide more reliable and accurate results than traditional classification algorithms. In this study, we propose a transfer learning based residual UNet architecture (TL-ResUNet) model, which is a semantic segmentation deep neural network model of land cover classification and segmentation using satellite images. The proposed model combines the strengths of residual network, transfer learning, and UNet architecture. We tested the model on public datasets such as DeepGlobe, and the results showed that our proposed model outperforms the classic models initiated with random weights and pre-trained ImageNet coefficients. The TL-ResUNet model outperforms other models on several metrics commonly used as accuracy and performance measures for semantic segmentation tasks. Particularly, we obtained an IoU score of 0.81 on the validation subset of the DeepGlobe dataset for the TL-ResUNet model.

## 1. Introduction

Most countries in the world, particularly European countries, have great agricultural potential. Some of the most important techniques that use machine and deep learning algorithms to achieve high productivity in precision agriculture include land cover classification and effective management of land resources. Numerous classifications of the physical coverage of the Earth’s surface, such as croplands, forests, grasslands, lakes, and wetlands are depicted on land cover maps as spatial information. Dynamic land cover maps incorporate transitions of land cover classes through time, thereby capturing changes in land cover. Land use maps provide geospatial information on the structures, activities, and resources that humans use to establish, enhance, or sustain a particular type of land cover.

More objects can now be identified in satellite images because of the rise in spatial resolution, and studies have switched from spectral image classification, pixel-based image analysis, and object-based image analysis to pixel-level semantic segmentation. In this study, we analyze the development of semantic segmentation techniques based on deep learning and propose a TL-ResUNet segmentation model for land use/cover.

In deep learning, many algorithms for classifying satellite images provide more reliable and accurate results than traditional classification algorithms, and numerous researchers are conducting various scientific and practical studies in this field [1,2,3]. Land use/cover maps are generated from different high-resolution satellite images, such as Sentinel [4], Landsat [5], and Worldview [6] satellite missions. These images can be used to classify different types of land cover, such as permanent water, built-up areas, residential areas, and agricultural fields. The Copernicus land monitoring service platform maintains general statistics on land use and cover across the world.

High-resolution satellite images have complex and deep features that require complex operations for image recognition. Creating land use maps is one of the most significant uses of satellite imagery, and this is possible through image segmentation and classification procedures. In recent years, different tasks and applications, such as producing regional and global land cover maps, creating advanced supervised and unsupervised classification algorithms, region-based image analysis, using numerous remote sensing features, and integrating map data into classification procedures, such as data on soil, roads, farmlands, crops, and other census data, have all seen significant advancements in the field of image classification. The main tasks of satellite image analysis are multi-object detection and classification that analyze numerical features or properties associated with an image, which can be divided into different classes. Comprehensive monitoring requires a highly productive evaluation of land cover via image segmentation and classification in different fields, particularly in agriculture.

Since 2012, CNN-based algorithms have been effectively used to solve classification tasks [7,8,9,10]. A set of convolution filters is used in each layer to identify image characteristics and features’ structure [11]. The most popular CNN-based architectures, such as GoogleNet, VGGNet, AlexNet, and ZFNet, have recently been used for image classification. However, calculating land use from satellite imagery through the classical approach of classification is difficult. Thus, segmentation-based classification has become significantly efficient and smart. Deep learning is a group of machine learning methods used in image analysis to learn and display features, such as edges, curves, and patterns from an input image. CNN and FCN are well-known deep learning techniques for image analysis. CNN-based structures include convolution, pooling, dropout, batch normalization, and non-linearity operation layers.

Therefore, this study presents a CNN-based UNet architecture, residual network, and transfer learning for land use classification of satellite images through semantic segmentation. Additionally, we discuss an overview of the recent deep learning-based techniques for satellite image classification and the available training datasets.

The main contribution of this work is improving model performance and accuracy using a combination of residual network, transfer learning, and UNet architecture. Generally, UNet is a robust architecture for segmentation tasks. Since land cover and land use classification task is complex, UNet coupled with residual networks and transfer learning yields better results.

The rest of this study is divided as follows: Section 2 analyzes various recent and relevant research papers; Section 3 studies available common datasets for satellite image segmentation and classification; Section 4 proposes our encode-decoder-based deep learning architecture (TL-ResUNet); Section 5 presents the experimental settings as well as the qualitative and quantitative analysis of the semantic segmentation results; and Section 6 presents the final remarks and conclusion of the study.

## 2. Related Works

Land cover has been studied in several research papers ranging from machine learning to deep learning. Using neural networks a decade ago was unpreferable because of their high computational complexity. Histogram thresholding provided satisfactory results, but exhibited problems associated with the variations and challenges in satellite images. Similarly, classical machine learning algorithms, such as support vector machines and random forest methods, were used for LULC mapping. For example, in [12,13] they have applied these methods for land cover classification. In the land cover classification study that uses machine learning, a decision tree and an artificial neural network were applied to Landsat ETM+ data to classify land cover. However, the drawback of these methods is that they require in-depth knowledge of the feature extraction process to improve model performance.

However, recent studies show that deep learning algorithms are widely used in classification and segmentation tasks. Especially, due to greater number of features and complex structure of satellite images, deep learning yields better results in LULC tasks such as agricultural field monitoring, forest change detection, water resources monitoring, building detection, and urbanization. For example, automatic building recognition method have been implemented in [14] which collected a dataset using the MapBox API for OpenStreetMap to create a satellite image with building masks. Furthermore, the pixel-wise image segmentation methods for classifying different attributes of satellite images is explained in study [15]. Here the proposed method can achieve a high accuracy using the UNet model to detect a building in the INRIA dataset, which is composed of very high-resolution images. However, these studies only focused on segmenting one class. Developing a model which uses multi-class segmentation of satellite images is a more complex task. Deep learning architectures, such as UNet and DenseNet, are actively used for image segmentation, whereas architectures, such as ResNet, VGG, and EfficientNet, are used for classification tasks in computer vision. According to the results of recent research works, these deep learning models outperform classical feature extraction algorithms.

However, in terms of satellite image processing, more work must be done to achieve high performance. For example, results of modern semantic segmentation were not satisfactory [16,17,18,19] due to the complex shape of satellite images. Kuo et al. [20] proposed a method that delivers one of the top results in the DeepGlobe challenge, in which improving the performance of model depends on a variation of DeepLabV3+. Despite this, their model accuracy is not good because of the fixed value of the standard deviation gaussian filter. Renee Su et al. proposed a semantic segmentation model using DeepLab v3+ with an IoU score of 0.756, and as a dataset they used the DeepGlobe dataset [21]. However, their model requires a greater number of satellite images to train because the authors did not apply any augmentation techniques. SegNet is a deep convolutional encoder-decoder architecture which is a very effective model among the numerous image segmentation models. Lee et al. applied the SegNet model to an aerial image to categorize the land cover and then performed research to assess the accuracy of that classification [22].

In [23], authors proposed an architecture using DeepLab and ResNet18 as the backbone, accomplishing an IoU score of 0.433 s of the DeepGlobe land cover data. The authors of the transfer learning approach in this study used two neural network architectures. The ResNet50 model was used for classification. After classification, a pre-trained ResNet50 model was used as an encoder in the modified UNet model for segmentation [24]. The accuracy was not so high, and the authors claim that this is mainly because of the quality of the dataset. Also, the authors conclude that the CORINE dataset is not suitable for training machine learning algorithms.

One of the main components of LULC is agricultural field monitoring. Several studies were conducted for farmland segmentation using low resolution images [25,26]. However, in [27] researchers generated a new benchmark dataset from VHR Worldview-3 images for twelve distinct LULC classes of two different geographical locations. Segmentation using low resolution satellite images can be used to classify tasks of global or general changes in areas, whereas high-resolution images should be used for segmenting specific objects such as multi-class segmentation and small objects.

## 3. Datasets

We collected publicly available satellite images for training and testing. However, the training dataset is constrained using this approach for satellite image classification and segmentation. To address this, we used image augmentation and various computer vision techniques to enhance the number of satellite frames. The shortage of labeled training data in a dataset has been one of the greatest challenges in adopting deep convolutional network pipelines in satellite image classification and segmentation. Datasets are created using middle or low-resolution satellite images. However, low and middle resolution satellite images may not produce the expected accuracy in satellite image segmentation. Pixel-based segmentation masks for image segmentation are considerably difficult to create. Applying a poorly supervised learning strategy, which is used in [28,29], is a method for tackling the lack of training data. The objective of weakly supervised methods is to reduce the need for complicated training datasets. Nivaggioli et al. [28] used a previously suggested method by producing pixel-level annotation from image-level annotation. They performed cropland segmentation using two types of labels commonly found in remote sensing datasets in [29]. To construct pixel-level maps of land cover, the study investigates weak labels in the form of a single-pixel label per image and class activation maps.

### 3.1. Labeled DeepGlobe Data

**Figure 1:**
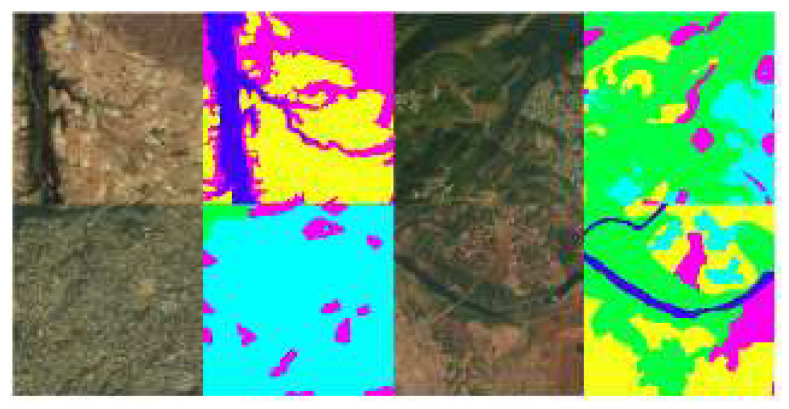
The land cover original image (**left**) and class label (**right**) pairs.

The DeepGlobe land cover classification challenge is the first publicly available dataset that focuses on rural regions using high-resolution submeter satellite images, as shown in Figure 1. The DeepGlobe dataset consists of approximately 1200 satellite images with a pixel size of 2448 × 2448, divided into training, validation, and test sets with a percentage of 70%, 15%, and 15%, respectively. Each image had RGB channels from the DigitalGlobe Vivid+ dataset with pixels at a resolution of 50 cm. Each satellite image was linked to a mask image to label the land cover. The mask is an RGB picture with seven classes, such as urban, agriculture, rangeland, forest, water, bare, and unknown (Table 1).

### 3.2. Defence Science and Technology Laboratory (Dstl) Dataset

The Dstl Kaggle dataset [30] is the second dataset, which provides 57 satellite images in a region of 1 sq. km. in both three-band RGB and 16-band multispectral formats. Here, we use three-band images with a spatial resolution of 1.24 m. In this dataset, 10 different classes, such as roads, buildings, vehicles, farms, trees, waterways, and others, have been labeled within particular images. The panchromatic waveband ranges from 450 to 800 nm, whereas 8 multispectral (red, red edge, coastal, blue, green, yellow, near-IR1, and near-IR2) wavebands are between 400 and 1040 nm. According to the sensor resolution at Nadir, panchromatic, multispectral, and SWIR bands are equivalent to 0.31, 1.24, and 7.5 m, respectively [31].

### 3.3. LandCoverNet

The multispectral satellite imagery from the Sentinel-2 mission in 2018 is labeled using the worldwide yearly LandCoverNet training dataset, as shown in Table 2. This dataset contains data across Africa, and each pixel of the image is identified as one of the seven land cover classes, such as water, woody vegetation, cultivated vegetation, semi-natural vegetation, permanent snow/ice, natural bare ground, and artificial bare ground, based on its annual time series.

The first version of this dataset contains 1980 images with a size of 256 × 256 pixels, which contains 66 tiles from the Sentinel-2. Each image chip includes an annual class label and temporal data from the Sentinel-2 surface reflectance product (L2A) at a 10-m spatial resolution, which is stored as a GeoTIFF data format. The resolution and an annual class label of each image are stored in a raster format, precisely as GeoTIFF files [32].

Table 2 compares datasets in terms of number of classes, spatial resolution, and number of images. While both Dstl and DeepGlobe are high resolution images, the latter was chosen for the proposed model because of the greater number of images. As mentioned earlier, during the experiments we found that image data augmentation approaches, such as geometric transformations, brightness/contrast enhancement, and data normalization, proved to be the most effective way to improve the final accuracy rate. The effectiveness of deep learning models depends on the size and resolution of the training image datasets. Therefore, we rotated each original image and then flipped each rotated image horizontally to increase the number of images in the satellite segmentation dataset. By applying the data augmentation methods to the original 3183 fire images, we increased the total number of images to 9700.

## 4. Proposed Architecture

**Figure 2:**
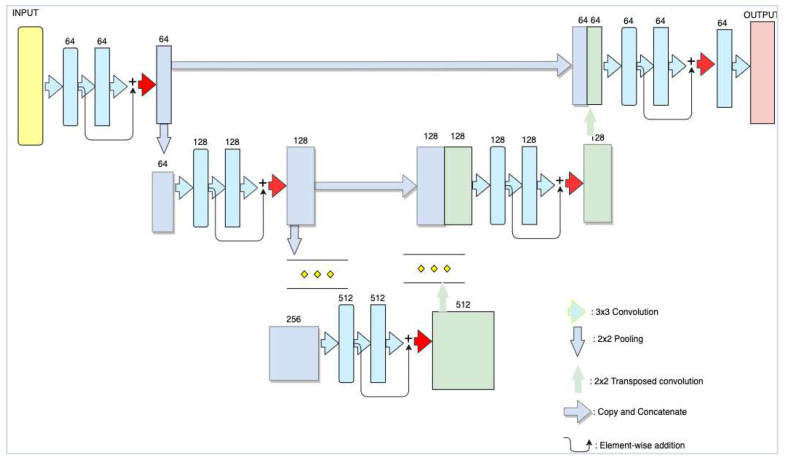
Modified UNet with a ResNet-50 encoder.

Two different neural network designs are suggested in this study. The first neural network architecture used for the segmentation task was the modified UNet model [33,34]. The second was the ResNet-50 model [35], which served both as the classification model and as an encoder for the modified UNet model, as shown in Figure 2. The UNet model was trained using different methods of ResNet backbone weight initialization models, that is, with random weights and ResNet pre-trained on the ImageNet dataset. With the help of this transfer learning strategy, we may apply the knowledge obtained from the first task to a new one, which is a more challenging task because obtaining training data is extremely difficult.

Additionally, the DeepGlobe dataset was used to train the satellite image segmentation model, which allowed for the use of ResNet weights that had already been learned, except for modifying and training the final layers of the network.

With regards to DeepGlobe dataset, it includes high resolution images with 1.24 m spatial resolution. The minimum requirements for the dataset is around 1000 high resolution satellite images, since deep learning models require greater number of images for training effectively. Using data augmentation techniques, the number of images in dataset increases during training model. The proposed model was trained using 9700 images.

### 4.1. Modified ResUNet Architecture

UNet is the most easily scalable and sizable fully convolutional network architecture for semantic segmentation. Generally, UNet architecture consists of two paths: a path that contracts to record context and another that expands symmetrically to enable exact localization. The contracting path follows a similar architecture to the ResNet architecture, where there is a long skip connection on every level; moreover, there are local skip connections between convolutions at each step. Feature maps are downsampled during the convolution processes, which also increases the number of feature maps per layer. However, they are upsampled before each step in the expanded route by a transposed convolution and this expanding branch boosts the resolution of the feature map. The expanding path uses skip connections to mix high-resolution features from the contracting path with upsampled features to localize them [35]. The output of the UNet model is a pixel-wise mask that shows the class of each pixel.

**Figure 3:**
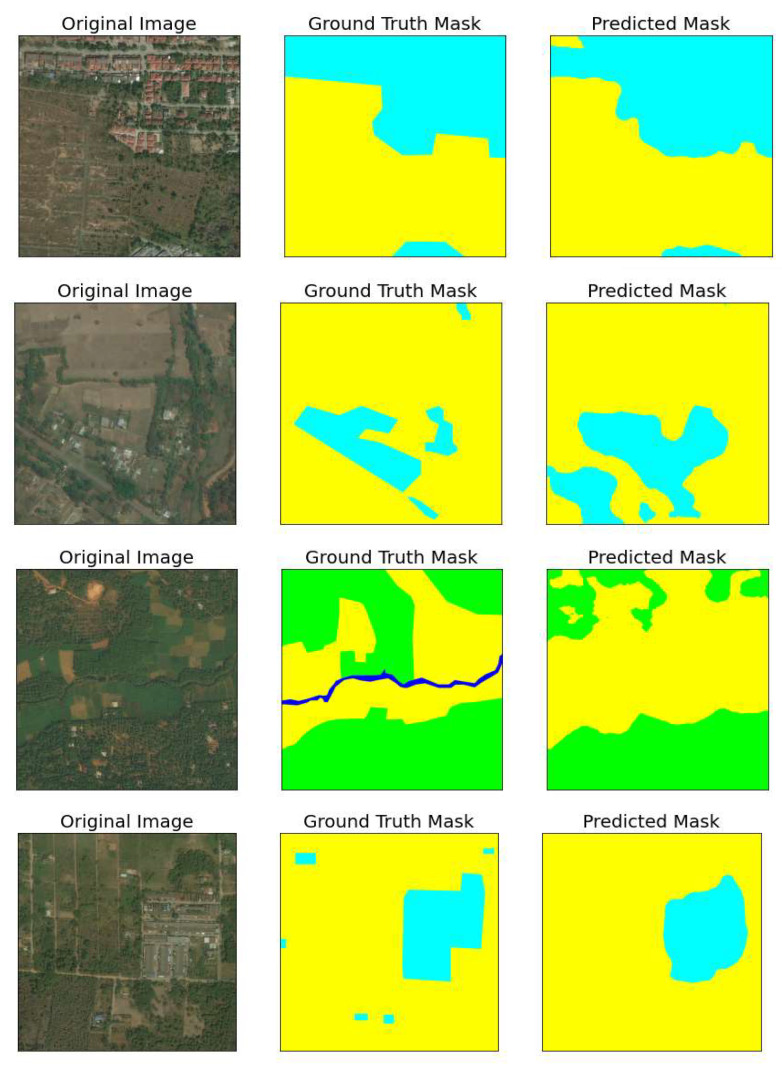

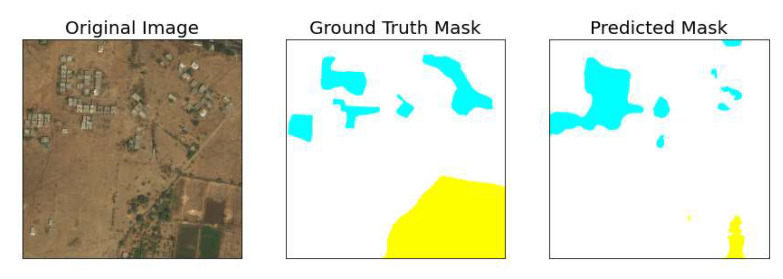
Trained model results.

We applied transposed convolution layers to build a matching decoder, which doubles the size of a feature map while cutting the number of channels in half. Then, the output of a transposed convolution is concatenated with an output of the corresponding part of the decoder. To maintain the same number of channels as in a symmetric encoder term, the resulting feature map is applied to a convolution process. Figure 3 shows that this upsampling process can be repeated several times to couple with max pooling layers. Technically, fully connected layers can accept inputs of any size, but because our max pooling layer downsamples each image twice, the present network implementation can only accept inputs with sides divisible by two.

### 4.2. ResNet Architecture

As an encoder of UNet, we used the pre-trained ResNet architecture, which consists of 48 convolution layers and 1 MaxPool layer, known as ReNnet-50. The advantage of ResNet over the sequential convolutional networks is that it can avoid the vanishing gradient problem and mitigate the degradation problem, where adding more layers to the model causes higher training errors. The ResNet architecture uses the repetitive layers of ResBlocks, that is, the blocks with skip connections, which make the network deeper while avoiding model degradation. After winning the ImageNet large-scale visual recognition contest for image classification in 2015, the ResNet architecture became recognized as the most sophisticated model architecture for image classification [35,36,37].

### 4.3. Evaluation Metrics

Both architecture models that modified UNet and ResNet-50 were assessed using the validation set, which consisted of 20% data. Key metrics, such as precision, recall, F1 score [38,39,40,41], and Jaccard index [42,43,44,45], were used to evaluate the model results. The precision metric was used to calculate the percentage of correctly labeled predictions across all predicted labels. It is the ratio of the true positive (TP) and false positive (FP) results (1):

(1) $$\mathrm{Precision}=\frac{TP}{TP+FP}$$

The *recall* metric was also considered to measure the proportion of correct labels in all predicted labels. It is the ratio of the TP and false negative (FN) results (2):

(2) $$\mathrm{Recall}=\frac{TP}{TP+FN}$$

The *F*_1_ score was used as a result of training the ResNet classifier, which combines precision and recall with the same weights (3):

(3) $$F_{1}=\frac{2\times \mathrm{precision}\times \mathrm{recall}}{(\mathrm{precision})+\mathrm{recall}}$$

The results of the UNet segmentation model were evaluated using the Jaccard index metric (4):

(4) $$J(A,B)=\frac{\left|A\cap B\right|}{\left|A\cup B\right|}=\frac{\left|A\cap B\right|}{\left|A\right|+\left|B\right|-\left|A\cap B\right|}$$

### 4.4. Loss Functions

We can modify the evaluation metric of the Jaccard index for discrete pixel-wise picture objects, where $y_{i}$ is the binary value (label) of the associated pixel and $y^{i}$ is the expected probability for the pixel. We used binary cross-entropy for the segmentation task since it can be viewed as a pixel-by-pixel classification problem. The area of intersection (J) between predicted masks and the related ground truth data is maximized by minimizing the loss function, which also optimizes the probability of correctly predicted pixels [46,47,48].

## 5. Experimental Results

The modified UNet model was used for semantic segmentation of the DeepGlobe dataset. The final modified TL-ResUNet model with initialized and tuned ResNet-50 encoder was trained and evaluated on the DeepGlobe dataset.

### 5.1. Model Training

The Pytorch framework is mostly recommended to train the machine and deep learning models. The modified UNet architecture is prototyped using the PyTorch framework by combining the building blocks of the ResNet-50 as an encoder. GPU servers with Nvidia Tesla V100 graphics cards and 43 GB of RAM were used during the training.

The ResNet-50 model is trained on the DeepGlobe dataset and initialized using weights of the ResNet-50 model pre-trained on the ImageNet dataset. The model is trained in two stages because a pre-trained model was used from the beginning: the first stage involves training just the last layers, whereas the second involves unfreezing all the layers. The model was trained with 20 epochs in total, i.e., 13 and 7 epochs for the first and second stages, respectively.

Three methods of weight initialization were considered during the training. First, the weights were initialized using a LeCun uniform initializer, which has a random uniform distribution within [−L, L], where L = sqrt (1/f_in_) and f_in_ is the number of input units in the weight tensor. Second, we reused the same architecture with the ResNet-50 encoder pre-trained on ImageNet, and all layers in the decoder were initialized using the LeCun uniform initializer. Third, we also used the latest trained segmentation model by initializing the encoder with the ResNet-50 but pre-trained on the DeepGlobe dataset, as shown in Figure 3.

### 5.2. Results

For the validation subset of the model using the DeepGlobe dataset, we achieved the following results after 30 training epochs:

(1) Best score on randomly initialized weights: IoU = 0.68;
(2) Best score on the encoder pre-trained weights on ImageNet: IoU = 0.81.

**Figure 4:**
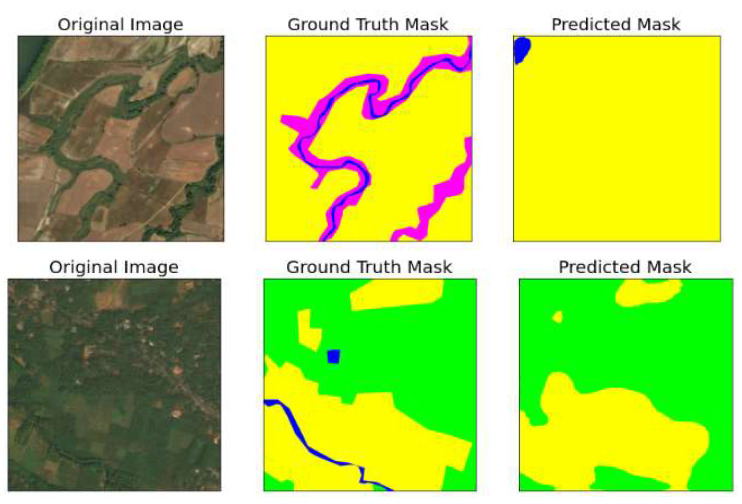
Examples of misclassification by the model.

Although the model performs well in the majority of cases (Figure 3), it may fail to detect some classes such as narrow water bodies. An example of this case is given below. Likewise, small, forested areas are misclassified in some cases (Figure 4). However, dense forested areas are classified correctly though they are located near agricultural fields. Distinguishing forested areas from farming lands is a challenging task. Furthermore, the model performs extremely good for some classes such as urban and farming lands.

**Figure 5:**
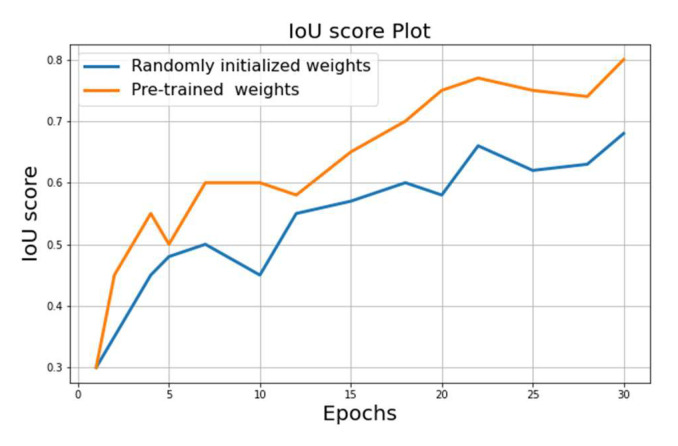

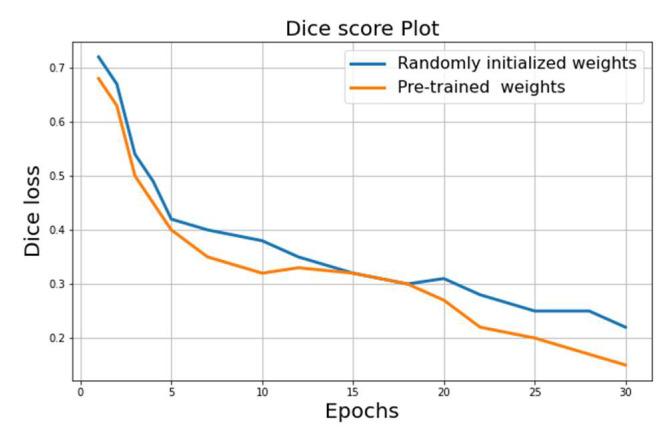
Learning curves of trained model at the training and validation stages.

The learning curves for validation in Figure 5 below show the results of each approach. A steady value is attained faster in pre-trained networks than in the randomly initialized network, and the steady value is visually higher in the pre-trained models.

The visualization of overlaying the masks on the original image demonstrates the advantage of training with the pre-trained models.

Note that the hyperparameter optimization techniques or the dataset preprocessing can be applied to further improve the performance of the models. Table 3 specifies the detailed scores.

The hyperparameter tuning techniques and results are shown in Table 3. Overall, it can be seen that UNet trained with transfer learning and residual layers can learn features faster and in an effective way. While UNet with random weights reaches a 0.68 IoU score in 30 epochs, UNet with ImageNet weights achieves a 0.74 IoU score. Finally, UNet with ResNet50 and with ImageNet weights achieved a 0.81 IoU score.

To further understand the advantage of our model against the others, we show some comparative results in Table 4. ClassmateNet produces fair segmentation output for larger areas but fails to segment short details such as smaller areas and field boundaries. DeepLabv3 and DeepLabv3+ improve performance on these details; however, they also produce artefacts and fail to keep producing stable results at larger areas in some cases. However, our model combines multi-level features effectively and produces more accurate segmentation results at both larger and detail areas.

We compared the robustness and weaknesses of previous methods with the proposed method in different categories using quantitative and qualitative performance results, as shown in Table 5. Based on the evaluated scores, the performance of the proposed approach did not suffer with densely forested areas and classified them correctly though they are located near agricultural fields. In addition, the model performs extremely well for some classes such as urban and farming lands.

The outcomes of segmentation methods can be divided into three categories: robust, standard, and powerless. Robust measures show that the method is applicable to segment all types of land/field segmentation. The algorithm may fail in some circumstances, such as narrow water bodies or small forested areas, according to normal standards. Powerless evidence suggests that algorithms are unreliable in the presence of noise or color, and the land classification procedure frequently modifies the initial geometry of moving objects.

## 6. Conclusions

In this study, we proposed a modified semantic segmentation deep neural network model called the TL-ResUNet for land use/cover classification and segmentation of satellite images. This developed model includes residual learning, UNet architecture, and a transfer learning approach. The proposed architecture section discussed the implementation of efficient training of the UNet model using pre-trained weights. The ResNet-50 model with pre-trained weights was chosen as a backbone of the UNet for experimental purposes. For the ease of building, training, and using the neural network, the library of the segmentation model, which is based on the PyTorch deep learning framework, was chosen. Finally, the environment and results of experimental training were analyzed using the commonly used IoU metric to determine the score of similarity of the predicted map and expected ground truth map. In the experiment, we verified the effectiveness of our proposed model and demonstrated that our model performs satisfactorily against the state-of-the-art models on the land use and cover task.

Future tasks include solving misclassification problems under similar color conditions and increasing the accuracy of the approach. We plan to develop a small real-time “land use land cover” model with YOLOv networks [49,50,51] using feature analyzing and extraction approach [52,53,54,55,56].

## Figures and Tables

**Table 1:** Classes in the label data of the DeepGlobe dataset.

Class/Color	Pixel Count	Proportion
Urban	642.4 M	9.35%
Agriculture	3898.0 M	56.76%
Rangeland	701.1 M	10.21%
Forest	944.4 M	13.75%
Water	256.9 M	3.74%
Barren	421.8 M	6.14%
Unknown	3.0 M	0.04%

**Table 2:** Comparison of main the characteristics of the abovementioned datasets such as DeepGLobe, Dstl and LandCoverNet.

Datasets	Number of Classes	Spatial Resolution	Number of Images
DeepGlobe	7	1.24 m	1146
Dstl	10	1.24 m	57
LandCoverNet	7	10 m	1980
Augmented Images	7	-	6517
Total	-	-	9700

**Table 3:** Comparison of the UNet training results on IoU metric throughout epochs.

Epoch	Train/Validation	UNet Trained with Randomly Initialized Weights	UNet Trained with Weights Trained on ImageNet without Residual Layers	UNet Trained with Weights Trained on ImageNet
10	train	0.51	0.52	0.54
	validation	0.49	0.50	0.51
20	train	0.59	0.62	0.69
	validation	0.58	0.61	0.64
30	train	0.68	0.75	0.84
	validation	0.68	0.74	0.81

**Table 4:** Comparison of the TL-ResUNet with other models.

Algorithms	IoU
Baseline	55.19
ClassmateNet	69.87
DFCNet	71.31
DeepLabv3	74.52
DeepLabv3+	75.6
TL-ResUNet	81.0

**Table 5:** Evaluation of the robustness and weaknesses of segmentation methods using different characteristics.

Criterion	DFCNet	DeepLabv3	DeepLabv3+	Proposed Method
Scene Independence	standard	robust	standard	robust
Object Independence	standard	robust	robust	standard
Robust to Noise	powerless	robust	standard	robust
Robust to Color	standard	standard	powerless	standard
Small Land Segmentation	robust	standard	robust	robust
Multiple Land Segmentation	standard	powerless	powerless	powerless
Processing Time	powerless	standard	robust	robust

## Data Availability

Data sharing not applicable.

## References

[1] Neupane B., Horanont T., Aryal J. (2021). Deep Learning-Based Semantic Segmentation of Urban Features in Satellite Images: A Review and Meta-Analysis. Remote Sens..

[2] Shafaey M.A., Salem M.A.M., Ebied H.M., Al-Berry M.N., Tolba M.F. (2019). Deep Learning for Satellite Image Classification.

[3] Alias B., Karthika R., Parameswaran L. Classification of high resolution remote sensing images using deep learning techniques. Proceedings of the International Conference on Advances in Computing, Communications and Informatics (ICACCI).

[4] Drusch M., Del Bello U., Carlier S., Colin O., Fernandez V., Gascon F., Hoersch B., Isola C., Laberinti P., Martimort P. (2012). Sentinel-2: ESA’s Optical High-Resolution Mission for GMES Operational Services. Remote Sens. Environ..

[5] Irons J.R., Dwyer J.L., Barsi J.A. (2012). The next Landsat satellite: The Landsat Data Continuity Mission. Remote Sens. Environ..

[6] Johnson K., Koperski K. (2017). WorldView-3 SWIR land use-land cover mineral classification: Cuprite, Nevada. Remote Sens. GIS.

[7] Scott G.J., England M.R., Starms W.A., Marcum R.A., Davis C.H. (2017). Training Deep Convolutional Neural Networks for Land–Cover Classification of High-Resolution Imagery. IEEE Geosci. Remote Sens. Lett..

[8] Musaev M., Khujayorov I., Ochilov M. Image Approach to Speech Recognition on CNN. Proceedings of the 2019 3rd International Symposium on Computer Science and Intelligent Control (ISCSIC 2019).

[9] Mukhamadiyev A., Khujayarov I., Djuraev O., Cho J. (2022). Automatic Speech Recognition Method Based on Deep Learning Approaches for Uzbek Language. Sensors.

[10] Valikhujaev Y., Abdusalomov A., Cho Y. (2020). Automatic Fire and Smoke Detection Method for Surveillance Systems Based on Dilated CNNs. Atmosphere.

[11] Kuchkorov T.A., Urmanov S.N., Nosirov K.K., Kyamakya K. (2020). Perspectives of deep learning based satellite imagery analysis and efficient training of the U-Net architecture for land-use classification. World Scientific Proceedings Series on Computer Engineering and Information Science, Developments of Artificial Intelligence Technologies in Computation and Robotics.

[12] Bengana N., Heikkilä J. (2021). Improving land cover segmentation across satellites using domain adaptation. IEEE J. Sel. Top. Appl. Earth Obs. Remote Sens..

[13] Tian C., Li C., Shi J. Dense fusion classmate network for land cover classification. Proceedings of the IEEE/CVF Conference on Computing and Vision Pattern Recognition Workshops 2018.

[14] Chhor G., Aramburu C.B., Bougdal-Lambert I. (2017). Satellite Image Segmentation for Building Detection using U-net. Comput. Sci. Semant. Sch..

[15] Karwowska K., Wierzbicki D. (2022). Improving Spatial Resolution of Satellite Imagery Using Generative Adversarial Networks and Window Functions. Remote Sens..

[16] Wafa R., Khan M.Q., Malik F., Abdusalomov A.B., Cho Y.I., Odarchenko R. (2022). The Impact of Agile Methodology on Project Success, with a Moderating Role of Person’s Job Fit in the IT Industry of Pakistan. Appl. Sci..

[17] Abdusalomov A., Mukhiddinov M., Djuraev O., Khamdamov U., Whangbo T.K. (2020). Automatic Salient Object Extraction Based on Locally Adaptive Thresholding to Generate Tactile Graphics. Appl. Sci..

[18] Sevak J.S., Kapadia A.D., Chavda J.B., Shah A., Rahevar M. Survey on semantic image segmentation techniques. Proceedings of the 2017 International Conference on Intelligent Sustainable Systems (ICISS).

[19] Huang G., Liu Z., Weinberger K.Q. Densely connected convolutional networks. Proceedings of the IEEE Conference on Computing and Vision Pattern Recognition 2017.

[20] Kuo T.S., Tseng K.S., Yan J., Liu Y.C., Wang Y.C.F. Deep aggregation net for land cover classification. Proceedings of the IEEE/CVF Conference on Computing and Vision Pattern Recognition Workshops 2018.

[21] Su R., Chen R. (2019). Land cover change detection via semantic segmentation. arXiv.

[22] Lee S., Park S., Son S., Han J., Kim S., Kim J. (2019). Land cover segmentation of aerial imagery using SegNet. Earth Resour. Environ. Remote Sens./GIS Appl. X. SPIE.

[23] Demir I., Koperski K., Lindenbaum D., Pang G., Huang J., Basu S., Hughes F., Tuia D., Raskar R. DeepGlobe 2018: A Challenge to Parse the Earth through Satellite Images. Proceedings of the 2018 IEEE/CVF Conference on Computer Vision and Pattern Recognition Workshops (CVPRW).

[24] Ulmas P., Liiv I. (2020). Segmentation of satellite imagery using U-net models for land cover classification. arXiv.

[25] Sharifzadeh S., Tata J., Sharifzadeh H., Tan B., Hammoudi S., Quix C., Bernardino J. (2020). Farm Area Segmentation in Satellite Images Using DeepLabv3+ Neural Networks. Data Management Technologies and Applications, DATA 2019.

[26] Kutlimuratov A., Abdusalomov A., Whangbo T.K. (2020). Evolving Hierarchical and Tag Information via the Deeply Enhanced Weighted Non-Negative Matrix Factorization of Rating Predictions. Symmetry.

[27] Sertel E., Ekim B., Osgouei P.E., Kabadayi M.E. (2022). Land Use and Land Cover Mapping Using Deep Learning Based Segmentation Approaches and VHR Worldview-3 Images. Remote Sens..

[28] Nivaggioli A., Randrianarivo H. (2019). Weakly Supervised Semantic Segmentation of Satellite Images. http://arxiv.org/abs/1904.03983.

[29] Wang S., Chen W., Xie S.M., Azzari G., Lobell D.B. (2020). Weakly Supervised Deep Learning for Segmentation of Remote Sensing Imagery. Remote Sens..

[30] Dstl Satellite Imagery Feature Detection. https://www.kaggle.com/competitions/dstl-satellite-imagery-feature-detection/data.

[31] Li Q., Shi Y., Huang X., Zhu X.X. (2020). Building Footprint Generation by Integrating Convolution Neural Network with Feature Pairwise Conditional Random Field (FPCRF). IEEE Trans. Geosci. Remote Sens..

[32] Alemohammad H., Booth K. (2020). LandCoverNet: A global benchmark land cover classification training dataset. arXiv.

[33] Ronneberger O., Fischer P., Brox T. (2015). U-net: Convolutional networks for biomedical image segmentation. arXiv.

[34] Nodirov J., Abdusalomov A.B., Whangbo T.K. (2022). Attention 3D U-Net with Multiple Skip Connections for Segmentation of Brain Tumor Images. Sensors.

[35] He K., Zhang X., Ren S., Sun J. Deep residual learning for image recognition. Proceedings of the IEEE Conference on Computing and Vision Pattern Recognition 2016.

[36] Kuchkorov T., Ochilov T., Gaybulloev E., Sobitova N., Ruzibaev O. Agro-field Boundary Detection using Mask R-CNN from Satellite and Aerial Images. Proceedings of the 2021 International Conference on Information Science and Communications Technologies (ICISCT).

[37] Kuchkorov T., Urmanov S., Kuvvatova M., Anvarov I. Satellite image formation and preprocessing methods. Proceedings of the 2020 International Conference on Information Science and Communications Technologies (ICISCT).

[38] Hossin M., Sulaiman M.N. (2015). A review on evaluation metrics for data classification evaluations. Int. J. Data Min. Knowl. Manag. Process.

[39] Abdusalomov A., Whangbo T.K. (2017). An improvement for the foreground recognition method using shadow removal technique for indoor environments. Int. J. Wavelets Multiresolution Inf. Process..

[40] Abdusalomov A., Whangbo T.K. (2019). Detection and Removal of Moving Object Shadows Using Geometry and Color Information for Indoor Video Streams. Appl. Sci..

[41] Farkhod A., Abdusalomov A., Makhmudov F., Cho Y.I. (2021). LDA-Based Topic Modeling Sentiment Analysis Using Topic/Document/Sentence (TDS) Model. Appl. Sci..

[42] Fletcher S., Islam Z. (2018). Comparing sets of patterns with the Jaccard index. Australas. J. Inf. Syst..

[43] Jakhongir N., Abdusalomov A., Whangbo T.K. 3D Volume Reconstruction from MRI Slices based on VTK. Proceedings of the 2021 International Conference on Information and Communication Technology Convergence (ICTC).

[44] Umirzakova S., Abdusalomov A., Whangbo T.K. Fully Automatic Stroke Symptom Detection Method Based on Facial Features and Moving Hand Differences. Proceedings of the 2019 International Symposium on Multimedia and Communication Technology (ISMAC).

[45] Kutlimuratov A., Abdusalomov A.B., Oteniyazov R., Mirzakhalilov S., Whangbo T.K. (2022). Modeling and Applying Implicit Dormant Features for Recommendation via Clustering and Deep Factorization. Sensors.

[46] Ayvaz U., Gürüler H., Khan F., Ahmed N., Whangbo T., Abdusalomov A. (2022). Automatic Speaker Recognition Using Mel-Frequency Cepstral Coefficients Through Machine Learning. CMC-Comput. Mater. Contin..

[47] Makhmudov F., Mukhiddinov M., Abdusalomov A., Avazov K., Khamdamov U., Cho Y.I. (2020). Improvement of the end-to-end scene text recognition method for “text-to-speech” conversion. Int. J. Wavelets Multiresolution Inf. Process..

[48] Khamdamov R., Saliev E., Rakhmanov K. (2020). Classification of crops by multispectral satellite images of sentinel 2 based on the analysis of vegetation signatures. J. Phys. Conf. Ser..

[49] Abdusalomov A., Baratov N., Kutlimuratov A., Whangbo T.K. (2021). An Improvement of the Fire Detection and Classification Method Using YOLOv3 for Surveillance Systems. Sensors.

[50] Mukhiddinov M., Abdusalomov A.B., Cho J. (2022). Automatic Fire Detection and Notification System Based on Improved YOLOv4 for the Blind and Visually Impaired. Sensors.

[51] Abdusalomov A.B., Mukhiddinov M., Kutlimuratov A., Whangbo T.K. (2022). Improved Real-Time Fire Warning System Based on Advanced Technologies for Visually Impaired People. Sensors.

[52] Abdusalomov A.B., Safarov F., Rakhimov M., Turaev B., Whangbo T.K. (2022). Improved Feature Parameter Extraction from Speech Signals Using Machine Learning Algorithm. Sensors.

[53] Khan F., Tarimer I., Alwageed H.S., Karadağ B.C., Fayaz M., Abdusalomov A.B., Cho Y.-I. (2022). Effect of Feature Selection on the Accuracy of Music Popularity Classification Using Machine Learning Algorithms. Electronics.

[54] Abdusalomov A., Whangbo T.K., Djuraev O. (2016). A Review on various widely used shadow detection methods to identify a shadow from images. Int. J. Sci. Res. Publ..

[55] Akmalbek A., Djurayev A. (2016). Robust shadow removal technique for improving image enhancement based on segmentation method. IOSR J. Electron. Commun. Eng..

[56] Farkhod A., Abdusalomov A.B., Mukhiddinov M., Cho Y.-I. (2022). Development of Real-Time Landmark-Based Emotion Recognition CNN for Masked Faces. Sensors.

